# Expression-Guided Deep Joint Learning for Facial Expression Recognition

**DOI:** 10.3390/s23167148

**Published:** 2023-08-13

**Authors:** Bei Fang, Yujie Zhao, Guangxin Han, Juhou He

**Affiliations:** 1 Key Laboratory of Modern Teaching Technology, Ministry of Education, Shaanxi Normal University, Xi’an 710062, China; beifang@snnu.edu.cn (B.F.); hgx@snnu.edu.cn (G.H.); 2 Department of Information Construction and Management, Shaanxi Normal University, Xi’an 710061, China; zhaoyujie@snnu.edu.cn

**Keywords:** facial expression recognition, deep joint learning, efficient CNN, expression-guided deep facial clustering, limited labeled data

## Abstract

In recent years, convolutional neural networks (CNNs) have played a dominant role in facial expression recognition. While CNN-based methods have achieved remarkable success, they are notorious for having an excessive number of parameters, and they rely on a large amount of manually annotated data. To address this challenge, we expand the number of training samples by learning expressions from a face recognition dataset to reduce the impact of a small number of samples on the network training. In the proposed deep joint learning framework, the deep features of the face recognition dataset are clustered, and simultaneously, the parameters of an efficient CNN are learned, thereby marking the data for network training automatically and efficiently. Specifically, first, we develop a new efficient CNN based on the proposed affinity convolution module with much lower computational overhead for deep feature learning and expression classification. Then, we develop an expression-guided deep facial clustering approach to cluster the deep features and generate abundant expression labels from the face recognition dataset. Finally, the AC-based CNN is fine-tuned using an updated training set and a combined loss function. Our framework is evaluated on several challenging facial expression recognition datasets as well as a self-collected dataset. In the context of facial expression recognition applied to the field of education, our proposed method achieved an impressive accuracy of 95.87% on the self-collected dataset, surpassing other existing methods.

## 1. Introduction

The face is the most commonly used characteristic for expression recognition [1] and personal identification [2]. In unobtrusive sensing, a camera sensor is a commonly used sensor, and the use of camera sensors for facial information capturing and recording is a commonly employed method. The advantage of using a camera to obtain face information is that it can be conducted without attracting the attention of the monitored subjects, thus avoiding their discomfort and interference. In addition, the camera can operate for a long time and monitor multiple scenarios and time periods, providing a large amount of face information data [3].

As one of the most fundamental tasks in face analysis, facial expression recognition (FER) plays an important role in understanding emotional states and intentions. FER also provides support in a variety of important societal applications [4,5,6], including intelligent security, fatigue surveillance, medical treatment, and consumer acceptance prediction. The FER methods attempt to classify facial images based on their emotional content. For example, Ekman and Friesen [7] defined six basic emotions based on a cross-cultural study, including happiness, disgust, surprise, anger, fear, and sadness. There have been various FER methods developed in machine learning and computer vision.

Over the past decade, image-level classification has made remarkable progress in computer vision. Much of this progress can be attributed to deep learning and the emergence of convolutional neural networks (CNNs) in 2012. The success of CNN inspired a wave of breakthroughs in computer vision [8]. However, while the deep CNN methods have become the most advanced solution for FER, they also have obvious limitations. In particular, a major disadvantage of deep CNN methods is their low sampling efficiency and the fact that they require a large amount of labeled data, which excludes many applications in which the data are expensive or inherently sparse.

In particular, annotating facial expressions is a complicated task. It is extremely time-consuming and challenging for psychologists to annotate individual facial expressions. Therefore, several databases use crowdsourcing to perform annotation [9]. For example, network datasets collected in uncontrolled environments, such as FER2013 and RAF-DB, have improved their reliability through crowdsourced annotations, but the number of annotated images is only about 30,000. The FER2013 database contains 35,887 facial expression images of different subjects, but only 547 of them show disgust. In contrast, deep learning-based approaches for face recognition are typically trained on millions of reliable annotations of face images. While the sizes of FER datasets are growing, they are still considered small from the perspective of deep learning, which requires a large amount of labeled data. For data-driven deep learning, the accuracy of direct training for such databases is low. Based on this observation, we refer to this problem as the limited labeled data obstacle; it inhibits the development of FER algorithms in deep learning. Therefore, determining how to recognize facial expressions efficiently and effectively from limited labeled data has become a critical problem.

For the FER methods relying on limited labeled data, there are two important strategies: transfer learning based on a face recognition model and semi-supervised learning based on large-scale unlabeled facial image data. One research stream focuses on applying transfer learning strategies to FER, i.e., fine-tuning deep networks on face recognition datasets to adapt them to the FER task [10]. Furthermore, another research stream focuses on applying semi-supervised learning-based deep convolutional networks to recognize facial expressions [11,12]. Two points indicate the potential for the application of semi-supervised learning strategies in FER: (1) Existing large-scale face recognition databases (such as the MS-Celeb-1M dataset [13]) contain abundant facial expressions; and (2) large amounts of facial expressions that are not labeled in databases, such as AffectNet and EmotioNet.

Motivated by the above observation and inspired by the above comments, in order to take full advantage of the limited labeled data and large amounts of unlabeled data, we conducted research on FER with limited labeled data and considered two concerns: the network structure and the training data. (1) Traditional CNNs generally have an excessive number of parameters and floating-point operations (FLOPs). However, the large parameter numbers make traditional CNN susceptible to over-fitting, especially when only limited labeled data are available. In order to improve the performance while reducing the number of parameters, we first proposed an efficient network from the perspective of limited labeled data. (2) Since a large-scale face recognition database contains an extensive number of facial expressions, the most feasible way to take full advantage of these numerous samples is to label them and then use these pseudo-labels as expression labels. Therefore, improving the efficiency and correctness of the pseudo-label labeling algorithm is another challenge to be solved in this paper. Using a pseudo-labeled training set and limited training data makes it possible to improve the accuracy of deep networks.

To address these problems, we propose an efficient FER approach based on a deep joint learning framework. In summary, this study primarily contributes in the following four ways.

- We develop an effective deep joint learning framework for FER with limited labeled data that can learn parameters and cluster deep features simultaneously by combining a deep CNN with deep clustering.
- We propose a novel efficient network, the affinity convolution-based neural network (ACNN), which greatly reduces the computational cost while maintaining the recognition accuracy. This network first generates a small number of intermediate features using convolution based on the affinity maps, and then applies a simple linear transformation to generate a large number of feature maps. This approach is more appropriate for limited training data.
- A new expression-guided deep facial clustering method is proposed to cluster deep facial expression features in face recognition datasets. This clustering algorithm is particularly efficient and suitable for large-scale clustering, with a low computational complexity and an efficient clustering performance.
- The experimental results of both inner-database evaluation and cross-database evaluation demonstrate that our framework surpasses existing state-of-the-art approaches. We investigate the factors that affect performance, including the influence of the network overhead, the impact of the clustering parameters, and we perform the visualization of deep features.

The rest of the paper is arranged as follows. We review relevant works on state-of-the-art FER methods and introduce FER with limited data in Section 2. The proposed deep joint learning framework for FER is introduced in Section 3. We present and discuss experimental results on four benchmark databases in Section 4 and Section 5. Finally, Section 6 summarizes the work and recommends future research directions.

## 2. Related Work

In this section, we first briefly review the development of facial expression recognition models, from traditional methods to deep-learning-based methods, and then we introduce FER with limited labeled data in more detail.

### 2.1. Efficient Network for Facial Expression Recognition

The existing FER methods that are described here use two distinct approaches, i.e., traditional FER and deep-learning-based FER. In traditional FER, handcrafted features are learned directly from a set of handcrafted filters based on prior knowledge. Traditional FER methods typically employ handcrafted features that are created using methods such as local phase quantization (LPQ) [14], histograms of oriented gradients (HOGs) [15], Gabor features [16], and the scaled-invariant feature transform (SIFT) [17]. As an example, Ref. [14] employed robust local descriptors to account for local distortions in facial images and then deployed various machine learning algorithms, such as support vector machines, multiple kernel learning, and dictionary learning, to classify the discriminative features. However, handcrafted features are generally considered to have limited representation power, and designing appropriate handcrafted features in machine learning is a challenging process.

Over the past decade, deep learning has proven highly effective in various fields, outperforming both handcrafted features and shallow classifiers. Deep learning has made great progress in computer vision and inspired a large number of research projects on image recognition, especially FER [18,19]. As an example, CNNs and their extensions were first applied to FER by Mollahosseini et al. [20] and Khorrami et al. [21]. Zhao et al. [22] adopted a graph convolutional network to fully explore the structural information of the facial components behind different expressions. In recent years, attention-based deep models have been proposed for FER and have achieved promising results [23,24].

Although CNNs have been very successful, due to the large amounts of internal parameters in CNN-based algorithms, they have high computing requirements and require a lot of memory. Several efficient neural network architectures were designed to solve the above problems, such as MobileNet [25] and ShuffleNet [26], which have the potential to create highly efficient deep networks with fewer calculations and parameters; they have been applied to FER in recent years. For instance, Hewitt and Gunes [27] designed three types of lightweight FER models for mobile devices. Barros et al. [28] proposed a lightweight FER model called FaceChannel, which consists of an inhibitory layer that is connected to the final layer of the network to help shape facial feature learning. Zhao et al. [29] proposed an efficient lightweight network called EfficientFace. EfficientFace is based on feature extraction and training, and it has few parameters and FLOPs.

Despite this, efficient networks are limited in terms of feature learning, because low computational budgets constrain both the depth and the width of efficient networks. Considering the challenges of pose variation and occlusion associated with FER in the wild, applying efficient networks directly to FER may result in poor performance in terms of both the accuracy and robustness. Furthermore, in a conventional lightweight network, such as MobileNet, pointwise convolution makes up a large portion of the overall calculations of the network, consuming a considerable amount of memory and FLOPs. Taking into account the above observation, we propose a new research contribution focused on developing an efficient neural network architecture based on the proposed affinity convolution that provides more discriminative features with fewer parameters for FER.

### 2.2. The Small-Sample Problem in Facial Expression Recognition

To mitigate the requirement for large amounts of labeled data, several different techniques have been proposed to improve the recognition results. In the following, we briefly review these approaches. Table 1 provides a comparison of the representative techniques for the small-sample problem.

(1) Data augmentation for facial expression recognition.

A straightforward way to mitigate the problem of insufficient training data is to enhance the database with data augmentation techniques. Data augmentation techniques are typically based on geometric transformations or oversampling augmentation (e.g., GAN). The geometric transformation technique generates data by maintaining the linear transformations of the label and performing transformations, such as color transformations and geometric transformations (e.g., translation, rotation, scaling) [35]. The oversampling augmentation technique generates facial images based on a GAN algorithm [30]. Although data augmentation is effective, it has a significant drawback, namely the high computational cost of learning a large number of possible transformations for augmented data.

(2) Deep ensemble learning for facial expression recognition.

In deep ensemble learning or the use of multiple classifiers, different networks are integrated at the level of features or decisions, combined with their respective advantages, and applied to emotional contests to improve their performance on small-sample problems [36]. Siqueira et al. [31] proposed an ensemble learning algorithm based on shared representations of convolutional networks; they demonstrated its data processing efficiency and scalability for facial expression datasets. However, it is worth noting that the ensemble learning methodology requires additional computing time and storage requirements because multiple networks (rather than a single learning category) are used for the same task.

(3) Deep transfer learning for facial expression recognition.

The transfer learning method is an effective method for solving the small-sample problem [37]. It attempts to transfer knowledge from one domain to another.

Fine-tuning is a general method used in transfer learning. Many previous studies have employed face recognition datasets like MS-Celeb-1M [13], VGGFACE2 [38], and CASIA WebFace [39] in order to pre-train networks like ResNet [8], AlexNet [40], VGG [41], and GoogleNet [42] for expression recognition. Then, these networks can be fine-tuned based on expression datasets like CK+, JAFFE, SFEW, or any other FER dataset to accurately predict emotions. For example, Ding et al. presented FaceNet2ExpNet [10], which was trained on a face recognition database and then trained on facial expressions and face recognition; it was fine-tuned on facial expressions to reduce the reliance of the model on face identity information. In spite of the advantages of training FER networks on face-related datasets, the identity information retained in the pre-trained models may negatively affect their accuracy.

Deep domain adaptation is another commonly used transfer learning method. This method uses labeled data from one or more relevant source domains to generate new tasks in the target domain [43]. To reduce dataset bias, Li et al. [1] introduced the maximum mean discrepancy (MMD) into a deep network for the first time. Taking advantage of the excellent performance of the GAN, the adversarial domain adaptation model [32] was rapidly popularized in deep learning for domain adaptation.

(4) Deep semi-supervised learning for facial expression recognition.

Semi-supervised learning (SSL) explores both labeled data and unlabeled data simultaneously in order to mitigate the requirement for large amounts of labeled data. Many SSL models have shown excellent performance in FER, including self-training models [33] and generative models [34]. The principles of SSL are based on a regularization-based approach to achieving high performance; however, they rely heavily on domain-specific data enhancements that are difficult to generate for most data modalities. Based on pseudo-label-based semi-supervised learning methods, a deep joint learning model is proposed. It alternates between learning the parameters of an efficient neural network and efficiently clustering and labeling facial expressions.

A study that is closely related to ours [12] suggested selecting high-confidence facial expression images to assist in network training with limited labeled data. Even though both our work and [12] propose labeling face recognition datasets and adding them to the training data for the network, our work differs in two key aspects: First, compared with [12], our proposed method not only solves the limited training data problem but also creates a more efficient network structure with fewer parameters and calculations. This makes it more suitable for small amounts of facial image training data and improves our FER performance. Second, in terms of labeling new data, Ref. [12] used knowledge distillation to compress the entire dataset into a sparse set of synthetic images. Unlike [12], the proposed expression-guided deep facial clustering method not only preserves the original features of the face data but also efficiently labels the data and controls the number of markers and their accuracy.

## 3. Methodology

### 3.1. Overall Framework

**Figure 1:**
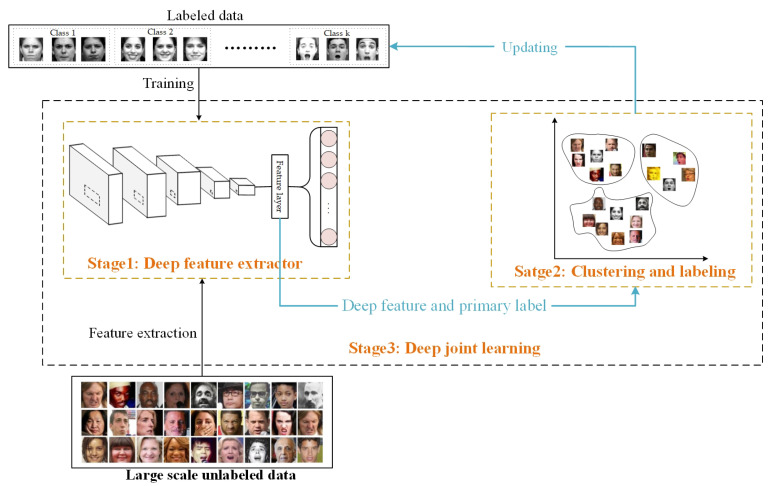
The proposed deep joint learning framework.

In this paper, a FER algorithm based on deep joint learning is proposed; it uses a small amount of labeled data and a rich unlabeled face recognition dataset. The proposed framework alternately learns the parameters of the efficient network based on an affinity convolution (AC) module structure (denoted as ACNN) and implements face clustering (denoted as EC) using a deep feature clustering assignment method guided by expressions. We call the whole deep joint learning framework ACNN-EC. The framework consists of a three-stage iterative learning procedure. It includes a deep feature extraction stage, clustering and labeling stage, and deep joint learning stage. We provide a description of the general procedure of ACNN-EC in Figure 1.

Before discussing our overall framework in detail, we first outline some preliminary aspects of FER formally. We are dealing with a facial expression dataset $D\;=\;\{D_{train},\;D_{test}\}$ and a face recognition dataset $D_{unlabeled}$. (1) First, we pre-train an efficient network model, ACNN, for facial expression recognition on $D_{train}$. At this stage, the pre-trained ACNN is used as the deep feature extractor, and $D_{train}$ and $D_{unlabeled}$ are classified. Then, both $D_{train}$ and $D_{unlabeled}$ have initial labels and corresponding deep feature representations. (2) In the second stage, the deep features of all training data ($D_{train}$ and $D_{unlabeled}$) are clustered, and expression-guided deep facial clustering is used. Then the confidence data and the corresponding pseudo-labels are selected. In the next iteration, the selected data will be added to the training dataset. (3) In the last stage, the pre-trained ACNN is fine-tuned using the selected pseudo-labeled data, as well as the labeled facial expression data, and a combined loss function is used to train the entire network model. The process continues iterating until the maximum number of iterations is reached or the error function is reduced to below some pre-set value.

### 3.2. Proposed ACNN for Facial Expression Recognition

#### 3.2.1. Proposed AC Module

Typically, deep learning-based approaches consist of a large number of parameters. In order to avoid problems such as over-fitting, the network requires a lot of training data. Although some recent studies have constructed efficient CNNs using shuffle operations [26] or depthwise convolution [25], the 1 × 1 pointwise convolution layers still consume the majority of the computational memory and FLOPs. To use cheap operations to generate more feature maps, we propose an efficient convolutional structure called the affinity convolution (AC) module. We first obtain a small number of more discriminative features from the weight generated by the affinity maps, and then we generate further features through inexpensive linear operations. In order to reduce the need for computing and storage resources, the AC module is used to add affinity maps to the primary convolution to produce a few intrinsic feature maps, and then it utilizes cheap linear operations to augment the features, as well as to increase the number of channels.

First, given the input feature map $X\;\in \;R^{h\;\times \;w\;\times \;c}$, *h* and *w* are the width and height of the input feature map, respectively, and *c* is the number of input channels. Specifically, we input the generated affinity maps $A\in \;R^{h\;\times \;w\;\times \;c_{A}}$ into the weight generation module *B* to generate the weight *W*:

(1) W=B(A)

inspired by [44], the weight generation module *B* consists of a standard convolutional layer, a normalization layer [45], and an activation layer (e.g., sigmoid). The number of channels $c_{A}$ in affinity maps is set to 4.

Second, the weight *W* is then inserted into the primary convolutional layer to generate the intrinsic feature maps:

(2) $$Y^{\prime }=\left(X\times W\right)*f^{\prime }$$

where $Y^{\prime }\in \;R^{h^{\prime }\times w^{\prime }\times m}$ represents the intrinsic feature maps, $h^{\prime }$ and $w^{\prime }$ are the width and height of the intrinsic feature map, *m* is the number of channels. The operation × between the input feature map *X* and weight *W* represents an element-wise multiplication operation. ∗ represents the convolution operation. $f^{\prime }\in \;R^{k\times k\times c\times m}$ represents the convolution filter used (m≤n), and the kernel size of the convolution filters $f^{\prime }$ is k×k. In practice, the primary convolution in the AC module here is pointwise convolution for its efficiency.

**Figure 2:**
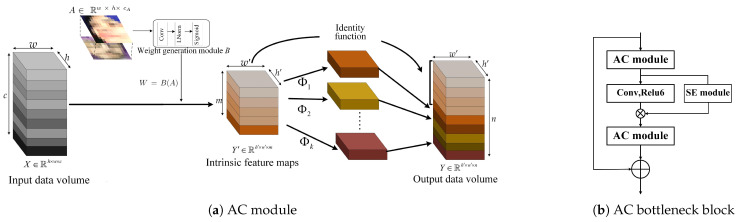
Proposed AC module and AC bottleneck.

Last, in order to obtain the *n* feature maps, *s* features are generated using a series of cheap linear operations that are performed on each intrinsic feature $Y^{\prime }$:

(3) $$y_{ij}=\phi_{i,j}\left(y_{i}^{\prime }\right),\forall_{i}=1,\ldots ,m,j=1,\ldots ,s,$$

where $\phi_{i,j}$ is the *j*-th linear operation used to generate the *j*-th feature map $y_{ij}$, and $y_{i}^{\prime }$ is the *i*-th intrinsic feature map in $Y^{\prime }$. Finally, the last $\phi_{i,s}$ is the identity mapping for preserving the intrinsic feature maps as shown in Figure 2a. In the implementation of linear operations, the group convolution is performed using linear operations. The AC module can obtain n=m×s feature maps $Y=[y_{11},y_{12},\ldots ,y_{ms}]$. With a set of intrinsic feature maps, the AC module generates a large number of feature maps at a low cost by using a series of parallel linear transformations.

#### 3.2.2. Proposed ACNN

Based on affinity convolution (AC), we propose an efficient neural architecture for FER. The proposed neural architecture is an efficient CNN based on an AC bottleneck block. First, we introduce the AC bottleneck block architecture in Figure 2b. Unlike in a conventional bottleneck block, a squeeze-and-excitation (SE) block [46] is inserted between the AC modules. The squeeze-and-excitation (SE) network, recognized as the winner of the ImageNet large-scale visual recognition competition (ILSVRC) 2017, has shown significant performance improvements for state-of-the-art deep architectures, albeit with a slightly higher computational cost. The SE block [44] is an innovative architectural unit designed to enhance the representational capacity of a network by enabling dynamic channel-wise feature recalibration [46]. Through the proposed AC bottleneck block, the percentage of pointwise convolution calculations in the overall network is reduced. Furthermore, the AC bottleneck block is also capable of performing multiple depthwise convolutions of different scales in order to extract the spatial features of an input feature map, thereby revealing more diverse and abstract information about the input feature map.

**Figure 3:**
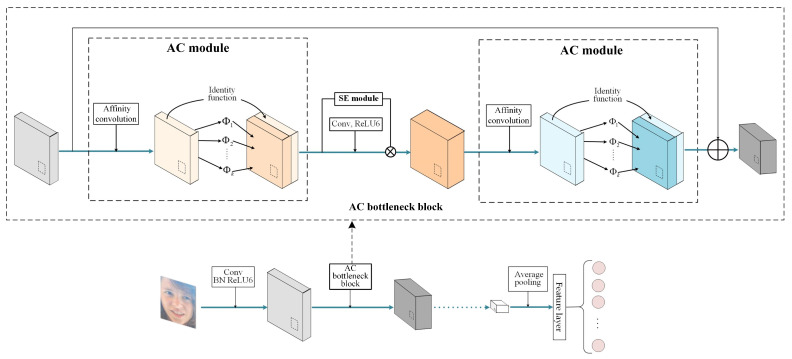
Overview of the proposed ACNN.

Based on the above AC bottleneck block, the state-of-the-art lightweight network MobileNet-V2 is used as the backbone network in our efficient CNN to reduce the computational overhead of the FER model; it is shown in Figure 3.

#### 3.2.3. Analysis of ACNN

In this part, we analyze the network from the number of parameters and FLOPs. We take the standard convolution process, given the input feature map $X\;\in \;R^{h\;\times \;w\;\times \;c}$, where *w* and *h* are the width and height of the input feature map, respectively, and *c* is the number of input channels; the standard convolution can be represented by the following equation:

(4) Y=X∗f+b

where $f\;\in \;R^{k\;\times \;k\times \;c\times \;n}$ represents the convolution filters, $Y\;\in \;R^{h^{\prime }\;\times \;w^{\prime }\;\times \;n}$ represents the output feature map, $w^{\prime }$ and $h^{\prime }$ are the width and height of the output data, respectively, *n* is the channel number, and ∗ represents the standard convolution operation. The kernel size of the convolution filters *f* is k×k. *b* is the bias term. In a standard convolution, the number of parameters and FLOPs involved can be calculated as follows:

(5) Parameters:k×k×c×n

(6) $$FLOPs:k\times k\times c\times n\times (h^{\prime }\times w^{\prime })$$

The number of parameters and FLOPs involved in the AC module can be calculated as follows. This calculation disregards the computational cost of the weight generation module. It is worth mentioning that pointwise convolution is performed in the primary layer, which involves element-wise multiplication of corresponding elements in the input tensor. The group convolution is performed as linear operations, where convolutions are performed within each group.

(7) $$Parameters:\left(k\times k\times m\right)+\left(m\times c\right)=\left(k\times k\times \frac{n}{2}\right)+\left(\frac{n}{2}\times c\right)$$

(8) $$\begin{array}{cc} FLOPs: & \left(k\times k\times m\times h^{\prime }\times w^{\prime }\right)+\left(m\times c\times h^{\prime }\times w^{\prime }\right)\\ \; & =\left(k\times k\times \frac{n}{2}\times h^{\prime }\times w^{\prime }\right)+\left(\frac{n}{2}\times c\times h^{\prime }\times w^{\prime }\right) \end{array}$$

Take a standard convolution with a 3×3×16×24 convolutional layer as an example, with an input volume of 112×112×16 and an obtained output volume of 112×112×24. The number of parameters is 3×3×16×24=3456 parameters, and 3×3×16×24×112×112= 43,352,064 FLOPs are required for the standard convolution. We replace the standard convolution with an AC module, which consists of a 1×1×12×16 primary layer and a 3×3×12×24 grouped convolutional layer. The number of parameters in the AC module is (3×3×12)+(12×16)=300 parameters. Furthermore, the AC module involves only (3×3×12×112×112)+(12×16×112×112)= 3,763,200 FLOPs. The proposed AC module has nearly 10 times less FLOPs computation than standard convolution.

### 3.3. Expression-Guided Deep Face Clustering

In order to eliminate the need for the manual annotation of data, facial expression clustering has received increased attention. There are some existing centroid-based clustering methods, such as k-means and fuzzy c-means clustering, which are widely used and easy to apply. Nevertheless, these methods are sensitive to the random initialization state, making their clustering results difficult to replicate. Furthermore, clustering large-scale deep facial features requires a more efficient clustering algorithm.

In recent research, the approximate rank-order clustering algorithm [47] has been shown to successfully overcome the challenges of large-scale clustering. The authors of [47] proposed an approximate rank-order metric for linking image pairs in order to define a cluster. The key concept of approximate rank-order clustering is to calculate the distance between only the first *k* nearest faces. Compared with other clustering methods, the computational complexity of approximate rank-order clustering is only O(kn), and its efficiency is much higher than that of other clustering methods. However, since approximate rank-order clustering is unsupervised, facial expression clustering is easily affected by face identity features, and it cannot utilize prior labeled facial expression information to guide the learning process. Based on the above analysis, we developed an expression-guided approximate rank-order clustering method, which effectively solves the problem of large-scale facial expression clustering.

For facial expression recognition, we propose an expression-guided semi-supervised deep clustering method based on an approximate rank-order metric. Specifically, based on the learned deep feature space, we develop a semi-supervised approximate rank-order clustering method to visually and semantically identify similar groups of facial expression images. Then, we re-annotate these groups with the initial labels obtained by ACNN to train FER classifiers. Next, we will discuss the process of the expression-guided deep facial clustering algorithm in detail.

**Figure 4:**
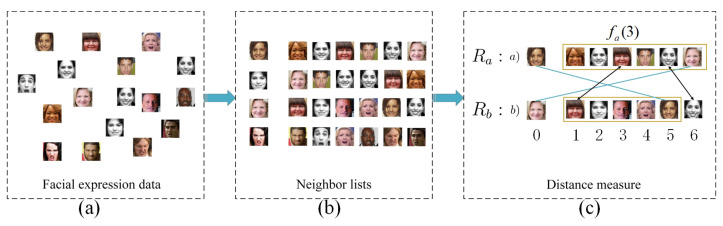
Expression-guided deep facial clustering. A set of facial expression images (**a**) is used to compute nearest neighbor lists (**b**); the nearest neighbor lists are then used to compute the distances between pieces of facial expression data (**c**).

First, we learn the deep features of the facial expression data from the deep feature extractor, as shown in Figure 4a. We measure the distance between two data according to their order in each other’s neighborhoods, i.e., the rank-order distance, as shown in Figure 4b. Second, we compute a series of top-*k* nearest neighbors in the facial expression data for the labeled expression data, as shown in Figure 4c. Depending on whether the data share nearest neighbors, the distance between data $x^{\left(a\right)}$ and $x^{\left(b\right)}$ is computed as follows:

(9) $$d\left(x^{\left(a\right)},x^{\left(b\right)}\right)=\sum_{i=a}^{min(R_{x^{\left(a\right)}}\left(x^{\left(b\right)}\right),k)}I_{x^{\left(b\right)}}\left(R_{x^{\left(b\right)}}\left(f_{x^{\left(a\right)}}\left(i\right)\right),k\right)$$

where $R_{x^{\left(b\right)}}\left(f_{x^{\left(a\right)}}\left(i\right)\right)$ represents the rank of the data $f_{x^{\left(a\right)}}\left(i\right)$ in the data $x^{\left(b\right)}$’s neighbor list, $f_{x^{\left(a\right)}}\left(i\right)$ represents the *i*-th data in the neighbor list of $x^{\left(a\right)}$.

(10) $$\begin{array}{c} I_{x^{\left(b\right)}}\left(\delta ,k\right)=\left\{\begin{array}{cc} 0 & sample\;\delta \;is\;in\;sample\;x^{\left(b\right)}’s\;top\;k\;nearest\;neighbors\\ 1 & otherwise \end{array}\right. \end{array}$$

where $I_{x^{\left(b\right)}}\left(\delta ,k\right)$ is an indicator function. $d(x^{\left(a\right)},x^{\left(b\right)})$ is an asymmetric distance function, this asymmetric distance function is further utilized to define a symmetric distance between two faces, $x^{\left(a\right)}$ and $x^{\left(b\right)}$ can be calculated as follows:

(11) $$D\left(x^{\left(a\right)},x^{\left(b\right)}\right)=\frac{d\left(x^{\left(a\right)},x^{\left(b\right)}\right)+d\left(x^{\left(b\right)},x^{\left(a\right)}\right)}{min(R_{x^{\left(a\right)}}\left(x^{\left(b\right)}\right),R_{x^{\left(b\right)}}\left(x^{\left(a\right)}\right))}$$

The pairwise distances between each pair of data can be computed using Equation (11). An illustration of expression-guided deep facial clustering is shown in Figure 4. In Figure 4c, we have two data samples, *a* and *b*. Using the Euclidean distance, we generate two neighbor lists, $R_{a}$ and $R_{b}$, by sorting the top six closest points. $f_{a}\left(3\right)$ represents the data that are the closest to sample *a*. From Figure 4c and the above equations, we can conclude that $R_{a}\left(b\right)=6$, $R_{b}\left(a\right)=5$, d(a,b)=3, d(b,a)=3, and D(a,b)=(3+3)/min(5,6)=6/5.

In the third step, we merge all data pairs whose distance is below a certain threshold. Finally, we label all data within the cluster with the same label as the vast majority of the data, based on the principle of majority rule.

### 3.4. Deep Joint Learning Based on Combined Loss Function

In spite of the fact that the two-stage methods could simultaneously learn deep feature representations and perform classification and clustering, their performance may be adversely affected by the errors accumulated during the alternation process. Our observations of the expression labels assigned by clustering based on deep facial features revealed that the clusters of different classes overlapped; for instance, the categories ‘disgust’ and ‘anger’ shared similar facial features, thereby contributing to inter-class similarities. Additionally, within each class, there are images whose facial expression intensities vary from low to high, thus resulting in intra-class variations. The results of [48] demonstrate that expression datasets are highly imbalanced in terms of inter-class similarities and intra-class variations. In contrast, Wen et al. [49] showed that the commonly used softmax loss was not significantly discriminative and could not ensure a high similarity for intra-class data or diversity for inter-class data.

According to the above analysis, in order to better classify and cluster the deep facial expression features learned by ACNN, we propose a combined loss function in which the center loss is combined with the additive angular margin loss [50]. The center loss is defined as

(12) $$L_{C}=\frac{1}{2}\sum_{i=1}^{m}{∥x_{i}-c_{y_{i}}∥}_{2}^{2}$$

where $x_{i}$ denotes the *i*th deep feature, belonging to the $y_{i}$th class, $c_{y_{i}}$ denotes the $y_{i}$th class center of the deep features by averaging over the deep features in the $y_{i}$th class. *m* is the training mini-batch, and the class centers are updated with respect to mini-batch *m*. This loss pulls closer training samples to their corresponding class centers.

The additive angular margin loss was first introduced by Deng et al. [50]; it improves the conventional softmax loss by optimizing the feature embedding on a hypersphere manifold where the learned expression representation is more discriminative. Suppose that $f_{i}$ denotes the embedding features computed from the last fully connected layer of the neural networks and $y_{i}=1,\ldots ,n$ is its associated class label. By defining the angle $\theta_{j}$ between $f_{i}$ and *j*th class center $w_{j}$ as $w_{j}^{T}f_{i}=∥w_{j}∥∥f_{i}∥\cos\theta_{j}$, the additive angular margin loss is defined as

(13) $$L_{A}=-\frac{1}{m}\sum_{i=1}^{m}\log\frac{e^{s\cos(\theta_{y_{i}}+a)}}{e^{s\cos(\theta_{y_{i}}+a)}+\sum_{j\neq y_{i}}e^{s\cos\theta_{j}}}$$

where *a* is the additive angular margin and *s* is the scaling parameter. The parameter setting of the loss function in the experiment uses the recommended parameters. More details about the optimization processes and recommended parameters can be found in [50].

The combined loss is shown as follows:

(14) $$L_{combined}=L_{C}+\lambda L_{A}$$

where $L_{C}$ and $L_{A}$ denote the center loss and additive angular margin loss, respectively. λ is a parameter that balances $L_{C}$ and $L_{A}$.

### 3.5. The Pseudocode of the ACNN-EC Framework

This section elaborates on the details of the proposed ACNN-EC for facial expression recognition. The specifics of our algorithm can be found in Algorithm 1. ACNN-EC comprises three main modules: deep feature extractor, clustering and labeling, and deep joint learning.
**Algorithm 1** ACNN-EC framework.**Require:** Labeled data $D_{train}$, unlabeled data (a face recognition dataset) $D_{unlabeled}$, test data $D_{test}$. Max iterations *N*.
**Ensure:** Label assignments for the test data $D_{test}$
 1:Input labeled data $D_{train}$, AC module is obtained by Equations (1), (2), and (3) respectively, and the ACNN network is constructed on the basis of the AC module. 2:Pre-train ACNN with combined loss by Equations (12)–(14). 3:**for** t=0 to N−1 **do** 4:    /*deep feature extractor:*/ 5:    pre-train(t=0)/fine-tune(t⩾1) DENet on $D_{train}$ 6:    **obtain** deep feature representations and initial labels for $D_{unlabeled}$ 7:    /*clustering and labeling:*/ 8:    cluster the deep features of all training data ($D_{train}$ and $D_{unlabeled}$) by Equations (10) and (11) 9:    **obtain** confidence data and the corresponding pseudo-labels by label comparison10:    **add** confidence data and their pseudo-labels to $D_{train}$11:**end for**12:**obtain** a label assignment on test data $D_{test}$

## 4. Experiments

To demonstrate the validity of our proposed method, ACNN-EC, a large number of experiments on two in-the-wild FER benchmarks, one lab-controlled dataset and our self-collected dataset, are performed. Then, cross-dataset experiments are performed on the CK+ dataset, MMI dataset, and self-collected dataset. The MS-Celeb-1M-v1c database is used as the facial expression clustering data, and the labeled data are used as pseudo-labeled data to augment the training data for the network. The FER datasets used in our experiments and the details of our implementation are described in the next subsection. Then, ACNN-EC is compared with several state-of-the-art approaches. In addition, experiments are performed to investigate the influence of each component of the proposed model.

### 4.1. Description of Databases

Experiments are performed on four FER datasets to evaluate the proposed algorithm, ACNN-EC. A more detailed description of the datasets is presented as follows. We obtained permission to use and display images from the face dataset described below.

(1) The RAF-DB database [9] contains 29,672 real-world facial images. There are two different emotion subsets in RAF-DB: single-label and multi-label. In this study, we adopt the single-label set, which contains 15,339 images, including 12,271 training images and 3068 test images.

(2) The FER2013 database [51] was obtained from the Google search engine. FER2013 includes 35,887 images with expression labels. These images consist of 28,709 training images, 3589 validation images, and 3589 test images.

(3) The extended Cohn–Kanade (CK+) database [52] consists of 593 video sequences from 123 subjects, and it is one of the standard benchmarks for FER. There are only 327 sequences that have been labeled with seven basic expression labels. Each of the final three frames of each sequence with peak formation is extracted, and the first frame (neutral face) is selected, yielding 1308 images labeled with seven basic expressions. Then the 1308 images are divided into 10 groups for n-fold cross-validation experiments.

(4) The MMI database [53] is a collection of 326 sequences, 213 of which have basic expression tags; it was constructed in a controlled laboratory environment. Compared with CK+, MMI includes onset-apex-offset labels.

(5) The MS-Celeb-1M-v1c database [54] is a celebrity recognition database. MS-Celeb-1M-v1c is a clean version of MS-Celeb-1M [13]. The original MS-Celeb1M consists of a large number of noisy faces. As a result, we use MS-Celeb-1M-v1c, which is clean but maintains the completeness of facial images. MS-Celeb-1M-v1c contains 86,876 identities and 3,923,399 aligned images. In this experiment, to reduce the face identity information, we extracted 86,876 identities from the whole database, each identity with 10 images.

(6) Our self-collected dataset was collected in an online learning environment, where we gathered facial expression information from learners. We recruited 10 university students, including 5 males and 5 females. The data collection was conducted in a laboratory environment, and in order to ensure that the online learning process of the learners was not disturbed, we provided them with a free and independent learning environment. The indoor lighting was not specially treated, and natural light from indoor fluorescent lamps and outdoor sunlight was used. Each student watched three ten-minute teaching videos, and a total of 30 videos of learners’ online learning were collected. Unlike datasets such as CK+ and MMI, which induce facial expressions, our dataset contains real emotional data in a natural environment. The video data were recorded with a camera (Logitech C1000e) to capture 2D facial motion data at 30 frames per second and 512 × 512 pixels.

**Figure 5:**
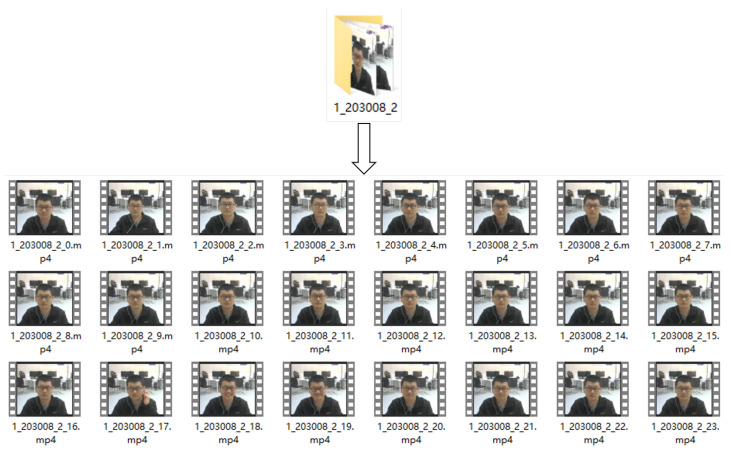
Example of video segmentation results.

After filtering out data that did not meet the experimental requirements, we obtained 1067 valid video segments, as shown in Figure 5. Referring to the processing method of the CK+ dataset, each of the final three frames of each sequence with peak formation is extracted, and the first frame (neutral face) is selected, yielding 71,185 images. These images were labeled in two ways: the first method involved labeling for the seven basic expressions, while the second method involved labeling for three levels of engagement (i.e., very engaged, nominally engaged, and not engaged) used to detect involvement. For this experiment, we only used data labeled for the seven basic expressions. The data were labeled through crowdsourcing. We used 56,947 images as training data, 7119 images as validation data, and another 7119 images as test data. The distribution of images among the seven emotions is shown in Table 2.

As can be seen from Table 2, there is a serious category imbalance in our self-collected dataset. In our experiments, we employed random flipping, random rotation, and random scaling operations to achieve approximate balance for each class. Although the number of subjects was relatively small, the number of training samples selected from the data of each subject was relatively large, with small intra-class distance and large inter-class distance.

### 4.2. Implementation Details

For all of the datasets used in our experiments, consistent with EfficientFace [29] and MA-Net [55], the face images are detected and aligned using RetinaFace [56]. It should be noted that the RetinaFace is robust to occlusions and non-front poses. Following this, the faces are all resized to 112 × 112 pixels. We initialize the proposed network, ACNN, with the weights that result when it is pre-trained on MS-Celeb-1M. The SGD is used as an optimizer, the mini-batch size is set to 128, the momentum is set to 0.9, and the weight decay is set to 0.0005. The initial learning rate is set to 0.001, and every ten epochs, it is multiplied by 0.1. For the combined loss, we set λ to 0.05. For clustering, we fix the distance thresholds *D* at 2.2, 1.9, 1.7, and 1.7 for the RAF-DB, FER2013, CK+, and self-collected datasets, respectively, and we set the number of node nearest neighbors in the four datasets to 8, 9, 5, and 7, respectively. Our reported results are based on an average of five-fold cross-validation experiments. Our method is implemented in PyTorch and runs using two NVIDIA 3080 Ti GPUs.

### 4.3. Results and Discussion

To evaluate the effectiveness of ACNN-EC, we adopt the most common FER settings, which are the inner-database evaluation and cross-database evaluation. The proposed method, ACNN-EC, is compared with various state-of-the-art deep learning methods. Our experiments are evaluated using the average classification accuracy. In the inner-database evaluation experiment, we evaluated our method on four FER benchmarks, namely RAF-DB, FER2013, CK+, and self-collected datasets. Table 3, Table 4, Table 5 and Table 6 summarize the FER accuracy on these four datasets, respectively.

Table 3 reports the performances of different models on the RAF-DB dataset. In Table 3, among all the competing models, [23], gACNN [24], and OADN [60] aim to disentangle the disturbing factors in facial expression images, and RAN and gACNN design a regional attentional branch network and assign different weights to facial regions according to the occlusion amount. SCN [61] was proposed to solve the noise label problem. DAS [12] and PAT [59] introduce more manual labels into the training data. Compared with algorithms that introduce more human labels into the training, such as the DAS and PAT algorithms, our algorithm achieves better results. MobileNet-V2 is employed as a baseline in our experiments, and we also consider some efficient CNNs, such as EfficientFace [29]. Compared with some FER algorithms based on an efficient CNN structure, the proposed ACNN-EC also has a higher recognition accuracy.

The performances of the models on the FER2013 database are reported in Table 4. Our method achieves an average recognition accuracy of 75.42%. We also compare it with DNNRL [64] and ECNN [65], where DNNRL uses InceptionNet as the backbone network and updates the model parameters according to the sample importance. ECNN proposes a probability-based fusion method to utilize multiple convolutional neural networks for FER. From Table 4, it can be seen that ACNN-EC obtains the highest recognition accuracy among the comparison methods, demonstrating its effectiveness and robustness for FER. In Table 5, for CK+, ACNN-EC achieved the highest recognition accuracy out of all the approaches.

Table 6 displays the inner-dataset experimental results on the self-collected dataset. It can be seen that our algorithm achieved excellent performance. Our self-collected dataset was used solely for inner-dataset experiments. However, we conducted five independent repetitions of the experiments to address any potential biases. This approach helped us mitigate optimistic bias and ensured a more robust evaluation of our proposed methods. It is worth noting that since the number of subjects was relatively small, and the number of training samples selected from each subject’s data was relatively large, the intra-class distance was small and the inter-class distance was large, so the overall accuracies of the algorithms were high in the self-collected dataset. In Section 5, we will conduct a more detailed quantitative evaluation of our self-collected datasets.

Cross-database experiments were carried out to test the generalizability of our model. For the cross-database evaluation experiment, we evaluated ACNN-EC on two lab-controlled datasets, namely CK+ and MMI. We trained the network on the RAF-DB database and tested it on the CK+ and MMI databases. The results of the cross-dataset experiments using CK+ and MMI are shown in Table 7 and Table 8. The results of previous studies were cited in [12]. It can be observed from Table 5 and Table 6 (ACNN-EC vs. WS-LGRN) and Table 8 (ACNN-EC vs. ECANN) that the performance gains of our proposed algorithm are moderate. However, it is important to note that our algorithm offers the advantage of reduced computational cost, which is another significant aspect highlighted in our experimental analysis. In particular, we examine the performance of ACNN-EC in terms of the number of parameters and FLOPs. By highlighting this advantage, we aim to provide a comprehensive understanding of the trade-offs between performance gains and computational costs when considering the application of our algorithm.

### 4.4. Empirical Analysis

In this subsection, the ACNN-module is analyzed on the RAF-DB dataset with a ResNet-50 backbone and a MobileNet-V2 backbone in terms of the number of parameters and FLOPs. Then, we investigate the impact of the hyperparameters on expression-guided deep facial clustering. Finally, we perform a visualization analysis of each iteration of the proposed deep joint learning model.

#### 4.4.1. Computational Overhead of Networks

In our experiments, the backbone network is either ResNet50 [8] or MobileNetV2 [63]. Then, expression-guided deep facial clustering is added to the network. Both ResNet-50 and MobileNet-V2 are pre-trained on MS-Celeb-1M. Table 9 shows the number of parameters and FLOPs and compares them with the baseline ResNet-50 and MobileNet-V2 models on the RAF-DB dataset.

Table 9 includes the proposed model trained with the two different backbone networks in the last two rows. As we can see, MobileNet-V2 provides more benefits to the model than ResNet-50. We analyze this because our proposed algorithm is more suitable for processing small sample data and avoids the overfitting that may occur with a large number of parameters in the case of ResNet-50. In addition, compared with the backbone network, the AC module structure in the lightweight network obtains a higher recognition rate; in addition, the number of parameters is significantly reduced, and the computational complexity is also greatly reduced. Our method achieves a high level of accuracy with a small number of computations; it exceeds the baseline by a large margin, from 83.96% to 90.98%.

In comparison with networks used in previous works, which often used ResNet or VGG-16 as the backbone network, our network is relatively small. Furthermore, compared with some FER algorithms based on an efficient CNN structure, such as EfficientFace [29] and MobileNet-V2 [63], although the proposed algorithm requires more computational resources and memory than EfficientFace, the proposed ACNN model has a better recognition accuracy. This is one of the main advantages of this method.

#### 4.4.2. Quantitative Evaluation of Expression-Guided Deep Face Clustering

In order to verify the effect of expression-guided deep facial clustering on the network training, we first investigate the performance of the expression-guided deep facial clustering method in the deep joint learning model on four FER datasets in Table 10.

As can be seen from Table 10, the number of initial labeled training images was relatively small, and the number of selected and labeled images was also relatively small, resulting in poor accuracy. However, by iteratively fine-tuning the network with the available labeled training data, the accuracy results were significantly improved. Specifically, starting from the second iteration of the model, we fine-tune the network with the newly labeled training data, and the accuracy improves to 87.29% and 90.98% on the RAF-DB dataset. It can be seen that ACNN-EC for expression-guided deep facial clustering effectively improves the network training and accuracy.

#### 4.4.3. Impact of Clustering Hyperparameters

Considering the importance of the expression-guided deep facial clustering method, it is important to investigate the impact of hyperparameters on expression-guided deep facial clustering. Here, we empirically set the clustering hyperparameters for the four datasets, as shown in Table 11. The distance thresholds for the clustering method were set to 2.2, 1.9, 1.7, and 1.7 for these four datasets, respectively. There is no formal method for selecting the most effective distance threshold, which is determined empirically. When dealing with extremely large datasets, such as the MS-Celeb-1M-v1c dataset, for example, one fundamental concern is the time cost, and the time cost is also an important evaluation metric. In Table 11, we see that based on the deep feature spaces generated for the four FER datasets, the running time for expression-guided deep facial clustering is satisfactory for large-scale face clustering. This also confirms our primary motivation for adopting this clustering algorithm.

#### 4.4.4. Deep Feature Visualization and Analysis

**Figure 6:**
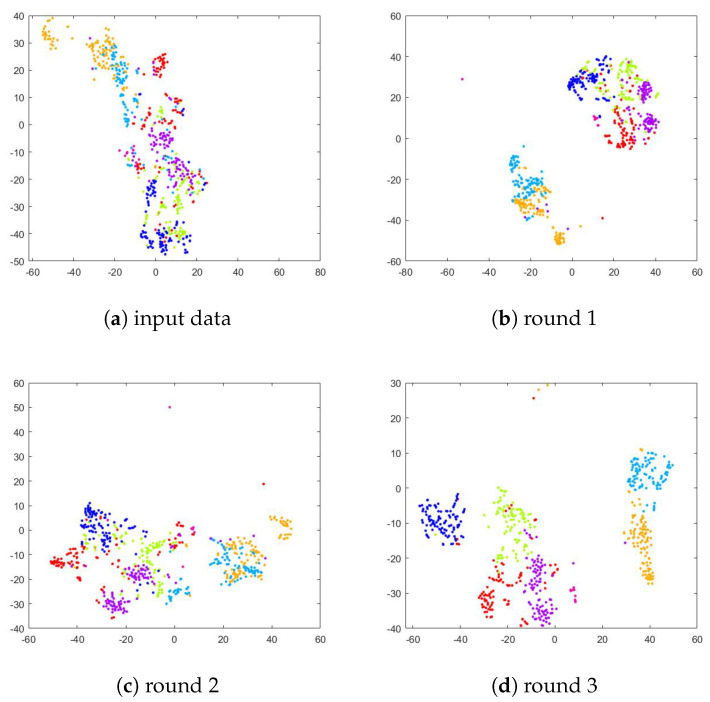
T-SNE visualization of extracted features from the RAF-DB dataset.

**Figure 7:**
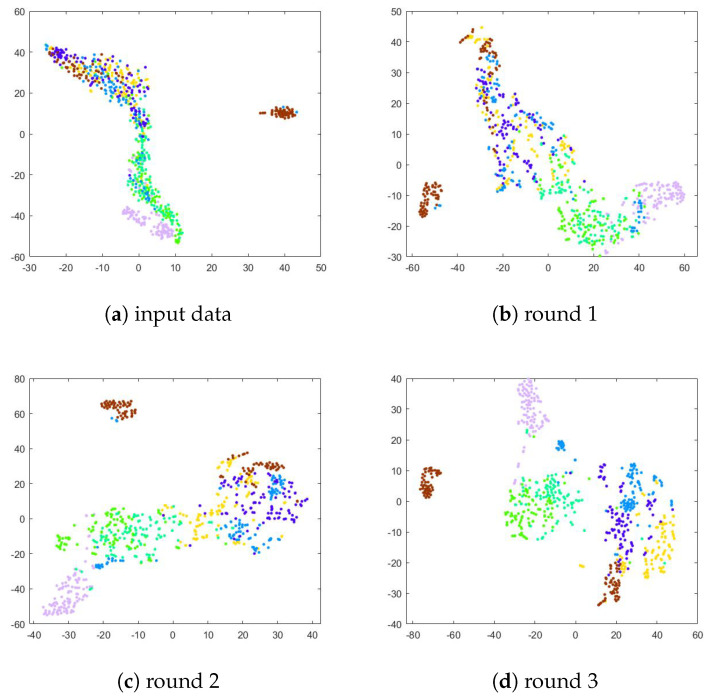
T-SNE visualization of extracted features from the FER-2013 dataset.

**Figure 8:**
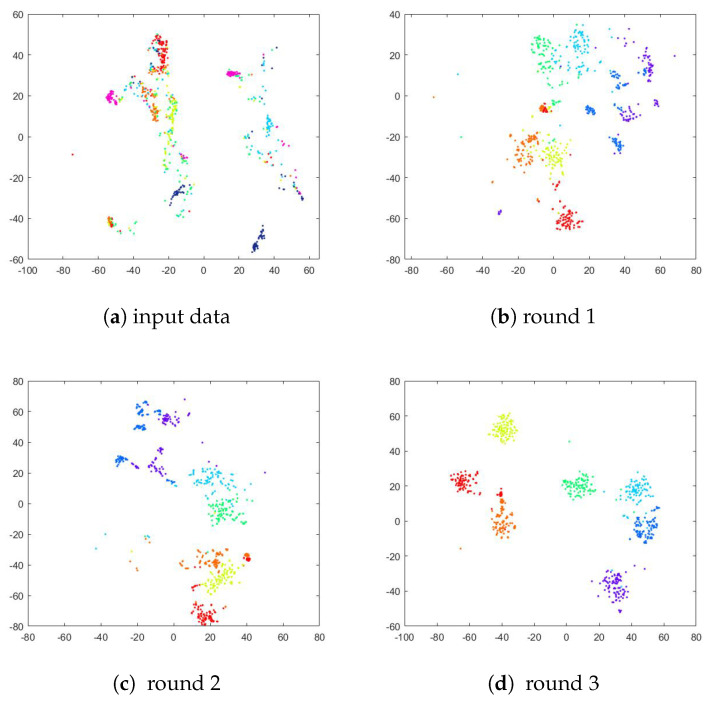
T-SNE visualization of extracted features from the CK+ dataset.

To verify the effect of the deep joint learning framework on the network training, we performed a comparison of the t-SNE [70] visualization results of the extracted features obtained in the original feature space and each iteration. Specifically, we extracted features from the images using the trained representation learner models. Following the training, we implemented t-SNE to visualize the projections of the extracted features to interpret our approach. As shown in Figure 6, Figure 7 and Figure 8, in the original feature space, the separability between different classes of data is poor because there are similar facial features among the face data. In the following iterations, the convergence of the data becomes more apparent. This distinguishable feature space not only improves the clustering performance but also facilitates the accuracy of the classification. Hence, it has been shown that ACNN-EC can better cluster data of the same class, demonstrating that it is able to learn more discriminative features for FER.

## 5. Quantitative Evaluation

### 5.1. Quantitative Evaluation Using Self-Collected Dataset

In this section, to validate the effectiveness of the proposed method in practical applications, we evaluated various classification methods using a self-collected dataset through rigorous quantitative evaluation procedures. Referring to evaluation metrics commonly used in previous research, such as widely adopted measures in object detection and classification, including accuracy, precision, recall, and F1 score, we conducted a comprehensive quantitative evaluation of the algorithm proposed in this paper. True positive (TP) represents the number of true positive samples, false positive (FP) represents the number of false positive samples, true negative (TN) represents the number of true negative samples, and false negative (FN) represents the number of false negative samples. Accuracy is the proportion of correctly classified samples in total samples, which is represented as the proportion of the sum of TP and TN to the sum of TP, FP, TN, and FN. Precision represents the proportion of the number TP to the sum of TP and FP. Recall represents the proportion of the number TP to the sum of TP and FN. The F1 score is the harmonic average value of precision and recall.

In Section 4.3, Table 6, we present an experimental analysis, where we compared our proposed algorithm with other popular methods in the field of facial expression recognition. Overall, our proposed algorithm achieved the highest classification performance, achieving an impressive accuracy of 95.87% on the self-collected dataset. It is worth noting that algorithms utilizing the ViT as a backbone, such as MVT [66], VTFF [67], and TransFER [71], also obtained competitive results, although not surpassing our method’s performance.

Table 12 presents the results of our proposed method on the self-collected dataset for seven different emotion categories, including quantitative evaluations of precision, recall, F1 score, and accuracy. From the table, it can be observed that the recognition accuracy for the “Neutral” and “Happy” categories is the highest, reaching 98.52% and 95.96%, respectively. We analyze that this is because the training sample set contains a large and diverse number of samples for the “Neutral” category, leading to high recognition accuracy. Additionally, the “Happy” category exhibits distinct facial expression features, and the facial expressions of happiness tend to be consistent across different individuals, contributing to the high recognition accuracy for this category. During the training phase, although we applied data augmentation to address the class imbalance issue in the self-collected dataset, traditional data augmentation affected the “Disgust” and “Fearful” categories differently. The recognition rate for the “Disgust” category reached 84.75%, while the recognition rate for the “Fearful” category was only 57.58%. Next, we will utilize the confusion matrices from the initial and final iterations to attempt an analysis of the underlying reasons.

### 5.2. Evaluation Based on Confusion Matrix

**Figure 9:**
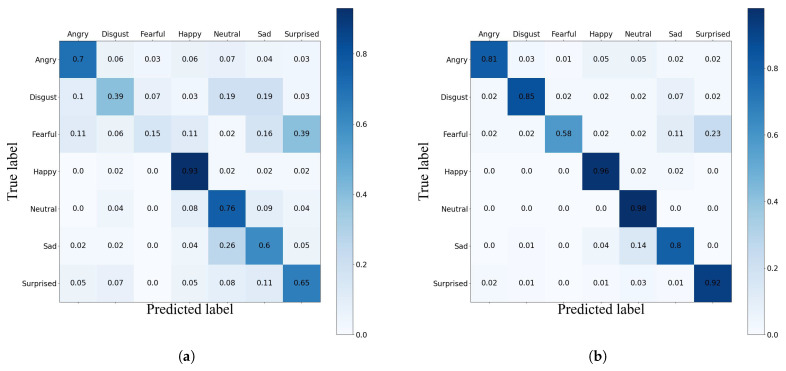
Confusion matrices for (**a**) the first iteration and (**b**) the last iteration on the self-collected dataset.

To comprehend the patterns of misclassifications within individual categories in the self-collected dataset, we present the confusion matrices for the initial and final iterations on the test set. From Figure 9, it can be observed that despite applying data augmentation techniques to the categories with fewer samples, the ’Fearful’ category had low accuracy in the initial iteration, which can be attributed to the lack of sample diversity and the difficulty of identification. However, a significant improvement was observed for this category after the addition of new samples. Furthermore, it can be concluded that the newly added samples had little impact on the classification of ‘Happy’ expressions. On the other hand, for the ‘Disgusted’ and ‘Surprised’ categories, which had low accuracy in the initial iteration, there was a significant improvement in the classification results.

### 5.3. Ablation Study on Using Self-Collected Dataset

To validate the insights provided by our algorithm into the role of each component in real-world applications, we conducted an ablation study to assess the contribution of individual modules. Table 13 illustrates the importance of each module of our method on the self-collected dataset. Model A indicates the algorithm’s direct use of the MobileNet v2 network to replace the proposed ACNN network. Model B represents the algorithm’s omission of using clustered labeled samples (EC) and directly undergoes one iteration. Model C demonstrates the algorithm’s use of Softmax Loss to replace the Combined Loss. Model D represents the ACNN-EC algorithm in our study. From Table 13 we observe that all components are helpful. The accuracy for Model A, Model B and-C was 89.97%, 75.08%, and 93.91%, respectively, while our algorithm achieved 95.87%, so we outperform by 5.9%, 20.79% and 1.96% margins. The algorithm’s performance is significantly enhanced by the clustering sample labeling technique, mainly due to the utilization of a substantial number of labeled samples, which greatly benefits the network training process.

### 5.4. Limitations and Discussion

In the context of facial expression recognition applied to the field of education, our proposed method achieved an impressive accuracy of 95.87% on the self-collected dataset, surpassing other existing methods. This high level of accuracy demonstrates the potential of our algorithm in enhancing emotion recognition systems for educational applications, thereby paving the way for more effective and personalized learning experiences for students. Although our method demonstrates excellent performance on our self-collected inner-dataset, we acknowledge the challenge of generalizing to previously unseen face data, especially those with limited samples. Fine-tuning the model on new datasets may not always yield optimal results due to domain shift and data scarcity. Additionally, the clustering accuracy may decrease when certain emotions are underrepresented or when there is a wide range of variations within an expression category.

In real-world applications, face images are typically not cropped, and there may be elements around the face such as hair, hats, or hands. Dealing with such high-resolution images indeed warrants exploration in applied research, and it is an area that we will focus on for future investigations. In addition, it will be crucial to evaluate the proposed method on real-world datasets with diverse environmental conditions, lighting, and facial expressions to validate its effectiveness in practical applications. Assessing the accurate recognition of emotions in the field of education is of paramount importance as it provides timely and tailored support to learners, ultimately leading to improvements in educational outcomes.

## 6. Conclusions

The central goal of this paper was to solve the problem of using deep learning methods to classify facial expressions when limited labeled data are available. This paper proposes a deep joint learning model, ACNN-EC, which combines a deep CNN with deep clustering. The model alternates between learning the parameters of an efficient neural network and efficiently clustering and labeling facial expressions. The AC-based CNN provides a better trade-off between the network performance and computational burden, with low-cost linear transforms for deep joint learning. The expression-guided deep facial clustering algorithm is an efficient algorithm designed specifically for large-scale clustering; it can effectively cluster facial expression features with low computational complexity. Furthermore, the experimental results and empirical analysis show that ACNN-EC is superior to other state-of-the-art approaches when a limited amount of labeled data is available. While our method is effective and the experimental results are encouraging, more could be done to achieve human-level performance with a small amount of data. Our future work may include improving the few-shot learning process by studying the network backbones and investigating more practical applications. In addition, considering the influence of other factors, such as race and skin color on facial expression recognition performance, is also a worthy direction of research [72]. In terms of practical applications, facial expression recognition algorithms can be made more meaningful by considering broader research applications, such as medical and health applications [6,72].

## Figures and Tables

**Table 1:** The comparison of representative facial expression recognition methods for the small-sample problem.

Method	Technique	Network	Datasets	Drawbacks and Advantages
[30]	Data augmentation	GAN	CK+, Oulu-CASIA, MMI, Multi-PIE, TFD	High computational cost
[31]	Ensemble learning	CNN	AffectNet, FER+	Additional computing time and storage
[10]	Fine-tuning	CNN	CK+, Oulu-CASIA, TFD, SFEW	The identity information retained in the pre-trained models may negatively affect accuracy.
[32]	Deep domain adaptation	GAN	BU-3DFE, KDEF, MMI	It requires access to many images in both source and target image domains at training time.
[33]	Self-training model	ResNet-18	KDEF, DDCF	They rely heavily on domain-specific data enhancements that are difficult to generate for most data modalities.
[34]	Generative model	GAN	CK+, Oulu-CASIA, BU-3DFE, BU-4DFE	Same as above

**Table 2:** Samples distribution for the self-collected dataset.

Emotion	Anger	Disgust	Fear	Happy	Sad	Surprised	Normal
Quantity	1981	458	1057	5741	1984	1892	43,834
Proportion	3.48%	0.8%	1.86%	10.08%	3.48%	3.32%	76.97%

**Table 3:** Inner-dataset experimental results on the RAF-DB dataset.

Method	Backbone	Accuracy (%)
FSN [57]	AlexNet	81.10
MRE-CNN [58]	VGG-16	82.63
gACNN [24]	VGG-16	85.07
PAT-VGG-F-(gender,race) [59]	VGG-16	83.83
PAT-ResNet-(gender,race) [59]	ResNet-34	84.19
OADN [60]	ResNet-50	87.16
SCN [61]	ResNet-18	87.03
RAN [23]	ResNet-18	86.90
PASM(3 rounds) [23]	ResNet-34	88.68
WS-LGRN [62]	DesNet	85.79
DAS [12]	ResNet-34	85.24
EfficientFace [29]	ShuffleNet-V2	88.36
MobileNet-V2(baseline) [63]	MobileNet-V2	83.96
ACNN-EC	MobileNet-V2	**90.98**

**Table 4:** Inner-dataset experimental results on the FER2013 dataset.

Method	Backbone	Accuracy (%)
DNNRL [64]	InceptionNet	71.33
ECNN [65]	Ensemble	69.96
PAT-VGG-F-(gender,race) [59]	VGG-16	72.16
PAT-ResNet-(gender,race) [59]	ResNet-34	72.00
PASM(2 rounds) [23]	ResNet-34	73.59
DAS [12]	ResNet-34	71.23
MobileNet-V2(baseline) [63]	MobileNet-V2	72.17
ACNN-EC	MobileNet-V2	**75.42**

**Table 5:** Inner-dataset experimental results on the CK+ dataset.

Method	Backbone	Accuracy (%)
Inception [41]	InceptionNet	93.20
PAT-VGG-F-(gender,race) [59]	VGG-16	95.58
PAT-ResNet-(gender,race) [59]	ResNet-34	95.82
DAS [12]	VGG-16	95.35
WS-LGRN [62]	DenseNet	98.37
MobileNet-V2(baseline) [63]	MobileNet-V2	94.24
ACNN-EC	MobileNet-V2	**98.95**

**Table 6:** Inner-dataset experimental results on the self-collected dataset.

Method	Backbone	Accuracy (%)
Inception [41]	InceptionNet	90.13
PAT-VGG-F-(gender,race) [59]	VGG-16	93.22
PAT-ResNet-(gender,race) [59]	ResNet-34	92.80
DAS [12]	VGG-16	91.62
WS-LGRN [62]	DenseNet	95.83
MobileNet-V2(baseline) [63]	MobileNet-V2	92.67
MVT [66]	Mask ViT	93.12
VTFF [67]	Hybrid ViT	93.01
TransFER [67]	Hybrid ViT	94.59
ACNN-EC	MobileNet-V2	**95.87**

**Table 7:** Cross-dataset experiment on the CK+ dataset.

Method	Backbone	Source	Target	Accuracy (%)
ECAN [68]	VGG-16	RAF-DB	CK+	86.49
gACNN [24]	VGG-16	RAF-DB	CK+	81.07
DAS [12]	VGG-16	RAF-DB	CK+	79.33
PASM [23]	ResNet-34	RAF-DB	CK+	79.65
RAN [23]	ResNet-18	RAF-DB	CK+	85.44
ACNN-EC	MobileNet-V2	RAF-DB	CK+	**89.68**

**Table 8:** Cross-dataset experiment on the MMI dataset.

Method	Backbone	Source	Target	Accuracy (%)
ECAN [68]	VGG-16	RAF-DB	MMI	69.89
gACNN [24]	VGG-16	RAF-DB	MMI	59.51
DAS [12]	VGG-16	RAF-DB	MMI	63.34
VGG-F(baseline)	VGG-16	RAF-DB	MMI	60.82
RAN [23]	ResNet-18	RAF-DB	MMI	58.29
ACNN-EC	MobileNet-V2	RAF-DB	MMI	**69.94**

**Table 9:** Comparison of results between the most advanced networks in terms of classification accuracy, the number of parameters, and FLOPs on RAF-DB.

Method	Backbone	#Params(M)	#FLOPs(M)	Accuracy (%)
gACNN [24]	VGG-16	>134.29	>15,479.79	85.07
RAN [23]	ResNet-18	11.19	14,548.45	86.90
SCN [61]	ResNet-18	∼11.18	∼1818.56	87.03
Res2Net-50 [69]	Res2Net-50	23.66	4278.6	88.19
ResNet-18 [8]	ResNet-18	11.18	1818.56	87.46
ResNet-50 [8]	ResNet-50	23.52	4109.48	87.72
EfficientFace [29]	ShuffleNet-V2	1.28	154.18	88.36
MobileNet-V2 [63]	MobileNet-V2	2.23	312.86	83.96
ACNN-EC(ResNet-50)	ResNet-50	11.76	2054.74	88.76
ACNN-EC(MobileNet-v2)	MobileNet-v2	1.56	196.43	**90.98**

**Table 10:** Analysis of the expression-guided deep facial clustering on RAF-DB, FER2013, CK+, and self-collected datasets.

	Round 1	Round 2	Round 3
Labeled training samples	12,271	42,743	137,869
Selected and labeled samples	30,472	95,126	∖
Accuracy (%)	78.52	87.29	90.98
Labeled training data	28,709	70,966	180,507
Selected and labeled data	42,257	109,541	∖
Accuracy (%)	69.87	71.29	75.42
Labeled training data	654	5834	35,769
Selected and labeled data	5180	29,935	∖
Accuracy (%)	85.69	95.21	98.95
Labeled training data	7119	27,324	115,483
Selected and labeled data	20,205	88,159	∖
Accuracy (%)	75.08	84.17	95.87

**Table 11:** Impact of clustering hyperparameters.

		Dataset		
	RAF-DB	FER2013	CK+	Self-Collected
Distance threshold *D*	2.2	1.9	1.7	1.7
Number of clusters *K*	235,540	267,517	226,981	206,312
Time cost	03:21:27	03:50:19	03:12:54	02:52:04

**Table 12:** The results of the evaluation metrics for the proposed method on the self-collected dataset.

	Precision (%)	Recall (%)	F1 score (%)	Accuracy (%)
Angry	96.17	81.05	87.96	81.05
Disgust	56.18	84.75	67.57	84.75
Fearful	95.00	57.58	71.70	57.58
Happy	92.98	95.96	94.45	95.96
Neutral	98.70	98.52	98.61	98.52
Sad	78.26	80.16	79.20	80.16
Surprised	77.03	92.37	84.01	92.37

**Table 13:** Ablation study, using the self-collected dataset.

	Model A	Model B	Model C	Model D
Accuracy (%)	89.97	75.08	93.91	95.87

## Data Availability

The data presented in this study are available in this article.
